# Differences in Functional Outcomes for Adult Patients with Prosthodontically-Treated and -Untreated *Shortened Dental Arches*: A Systematic Review

**DOI:** 10.1371/journal.pone.0101143

**Published:** 2014-07-03

**Authors:** Saadika Khan, Alfred Musekiwa, Usuf M. E. Chikte, Ridwaan Omar

**Affiliations:** 1 Department of Restorative Dentistry, University of the Western Cape, Cape Town, South Africa; 2 Centre for Evidence-Based Health Care, Faculty of Medicine and Health Sciences, Stellenbosch University, Cape Town, South Africa; 3 Department of Interdisciplinary Health Sciences, Stellenbosch University, Cape Town, South Africa; 4 Head of Prosthodontics, Faculty of Dentistry, Kuwait University, Safat, Kuwait; UNC School of Dentistry, University of North Carolina-Chapel Hill, United States of America

## Abstract

This review examined differences in functional outcomes and patient satisfaction when shortened dental arches are left untreated compared to their restoration to complete arch lengths with different prosthodontic interventions.

**Methods:**

A protocol was developed according to the criteria for a systematic review. All relevant databases were searched to identify appropriate clinical trials regardless of language or publication status. Predetermined eligibility criteria were applied, trial quality assessed and data extracted for each study. Relevant outcomes assessed were: functioning ability, patient satisfaction and harmful effects on oral structures.

**Results:**

Searches yielded 101 articles: 81 from electronic databases and 20 from reference lists of retrieved articles (PEARLing searches). Sixty-nine citations were assessed for eligibility after removing 32 duplicate records. After reading titles and abstracts, a total of 41 records were excluded and the full-texts of the remaining 28 records were read. Only 21 records were included for the SR because 7 records were excluded after reading the full-text reports. These 21 records report the outcomes of four randomized controlled trials (RCTs) and one non-randomized clinical trial (CT) which were pre-specified and used for this review. No on-going studies were found and no eligible studies were excluded for failure to report the reviewer’s pre-specified outcomes. Outcomes were reported in the retrieved 21 articles. A narrative explanation of the pre-specified outcomes is reported for the 3 comparison groups (which were based on the different interventions used for the individual clinical trials). The shortened dental arch as a treatment option is encouraging in terms of functioning, patient satisfaction and cost-effectiveness. By using only high quality studies it was expected that the results would be more reliable when making conclusions and recommendations, but some of the included studies had to be downgraded due to methodological errors.

## Introduction

Prosthodontic treatment planning customarily includes the replacement of all missing teeth with the intention of achieving complete dental arches (CDAs) comprising 28 teeth [1]–[3]. The rationale for this approach includes impaired oral function with a perceived detrimental impact on chewing ability, occlusal stability and temporomandibular joint (TMJ) function due to the loss of the molar teeth [4]. On the other hand, several studies and reviews have indicated that twenty occluding teeth provide sufficient oral functional ability and the need to replace all missing posterior teeth has been questioned [3]–[11].

The classic shortened dental arch (SDA) is defined as ten pairs of occluding anterior and premolar teeth [5], [8]. Many patients present with SDAs since molars are the teeth more commonly lost due to caries, resulting in patients having a posteriorly reduced dental arch [12]–[13]. Variations of the SDA include a partially dentate arch described as an interrupted or discontinuous dental arch where individual anterior, premolar or even molar teeth are lost [7]. A considerable number of studies have been conducted, though mostly in industrialized countries, that confirm a range of benefits and adequate oral functioning with a SDA [4]–[12], [14]–[20]. These studies also propose that the aesthetic features of such partially dentate patients are acceptable [5], [8]. Research related to the SDA concept has also been conducted and promoted in some developing countries such as Tanzania and Nigeria [3], [5]–[12]. The 1982 WHO oral health goal for developing countries was set as the retention of twenty functional, aesthetic natural teeth without resorting to a prosthesis which is in line with the findings of the SDA research [4]–[12], [21].

When dentists extend or reconstitute reduced, shortened or discontinuous dental arches and replace missing teeth in either anterior or posterior regions to create a CDA, the following interventions are usually recommended: removable partial denture prosthesis (RPDP) or fixed denture prosthesis (FDP), including resin-bonded bridges and implant-retained prostheses [9], [22]–[33]. Anecdotal evidence suggests that the choice is largely intuitively based upon the number of missing teeth, their location in the arch, and economic considerations. Currently, RPDPs, FDPs and implant procedures evidently operate on the premise of optimal occlusion encompassing the aesthetics, oral function, oral health and comfort created by the occluding teeth [4]–[5], [33]. This practice appears to have evolved empirically, with no scientific or clinical evidence to support its widespread acceptance by clinicians [3], [22]–[33].

Several research reports tend to support the view that the underlying objective of the SDA to preserve a functional dental arch can be realized through a functionally-oriented treatment approach [5], [15]–[17], [22], [24], [26]. This entails directing the limited resources towards that part of the dentition that can be successfully preserved and in the most cost-effective manner, rather than on the remaining molar teeth that often have a poorer prognosis [5], [7], [31]–[58]. The minimum number of teeth or shortness of the arch will also depend on the periodontal condition of the remaining teeth, the age of the patient, occlusal activity, food types and adaptive capacity of the patients’ temporomandibular joints [3], [7], [9].

Research suggests that this seemingly beneficial SDA concept and its variations can be utilized to improve accessibility and affordability to treatment for socially- and economically-deprived middle-aged and elderly communities [5], [16], [22], [24], [26]. Other associated benefits of the SDA have been enumerated by several researchers [5]–[8], [10]–[20], [31]–[58]. A number of studies have been conducted in Tanzania where the evidence obtained was used to advise the government, medical and dental personnel to include the SDA concept within the prosthodontic management protocols for the country [12], [16], [50]–[52]. The consequence of the research was that dental institutions reviewed the dental curricula accordingly [12], [16], [50]–[52].

Following the large body of published research data related to the SDA conducted in different parts of the world, several efforts at collating these data have been made. Thus a number of systematic reviews (SR) focusing on the SDA have been completed [8], [56]–[58]. A SR conducted by Gotfredsen and Walls (2007) focused on studies that reported on the assessment of normative needs only, although it did not include quality of life studies that considered the perceived oral health needs of partially dentate patients [8]. In the SR by Fueki et al (2011), different types of study designs were included, in addition to the randomised controlled trials (RCT) [56]. The quality of evidence from longitudinal studies related to restorative and non-restorative approaches to adult patients with SDAs were assessed by Faggion (2011) using GRADE (Grading of Recommendations Assessment, Development and Evaluation) approach [58]. With this study, even though all the results from the included studies were not reported, it demonstrated how important methodological rigor is and that these need to be reported [58]. In a recent electronic search in the Cochrane database for systematic reviews, Abt, Carr and Worthington (2012) focused on a broad research question to include all different types of interventions for partially dentate patients, including the SDA [57]. No conclusive evidence was found to indicate that any intervention was better for partially dentate patients, irrespective of particular interventions, procedures or materials used [57].

Given that so few RCTs have been conducted and are available, researchers conducting SRs are faced with the ineluctable option of including different types of study designs and systematic reviews [8], [56]–[58]. This results in the inclusion of lesser strength studies which could affect the quality of the evidence presented [8], [14], [31]–[37], [40]–[48], [53]–[55].

The aim of this systematic review was to identify and analyse existing clinical trials which compare the functional outcomes of prosthodontic interventions used for treating shortened arches versus un-restored shortened arches in partially dentate adult patients.

The following research question addresses the aim and objectives of the study: In adult patients with shortened dental arches, what is the effect of prosthodontics interventions on the functional outcomes compared to having no treatment?

## Methods

### Protocol Development

A protocol (Registration No: 11/4/39) was developed (not published) to include all aspects of a SR namely: selection criteria, search strategy, selection methods using predetermined eligibility criteria, data collection, data extraction, assessment of risk of bias using the Cochrane tool, the GRADE tool to grade the evidence of each clinical trial and statistical analysis by calculating risk ratios (RR) for dichotomous outcomes and presented at 95% confidence intervals [59]–[60].

### Criteria for considering studies for this review

#### Types of studies

Only RCTs and Clinical trials (CTs) are included in the systematic review (SR).

#### Types of interventions

Interventions included in this study are described as any prosthodontic intervention used to restore and treat the SDA such as RPDPs and FDPs. The control group for this study included patients with the classic SDA.

#### Types of participants

Participants included in the SR were:

1. Adult male and female participants aged 18 years and older.
2. Study population included patients with posteriorly reduced or shortened dental arches.

#### Types of outcome measures

Primary and secondary outcomes were pre-specified for the SR and these include:

#### Primary outcomes

1. Functional outcomes (patient- or investigator-reported) as measured by masticatory function, chewing ability, occlusal effects, nutrient intake (using nutritional assessments and haematological markers) and subjective functioning ability.
2. Survival of the interventions (fixed or removable partial denture prostheses) used for the extension of SDAs.

#### Secondary outcomes

1. Patient satisfaction and oral health-related quality of life (social interaction; aesthetics and effectiveness) using oral health indicators for example Oral Health Indicator Profile (OHIP) or the Oral Impacts of Daily Performance (OIDP).
2. Harmful effects (caries; tooth loss; periodontal status, plaque index (PI), gingival index (GI), temporomandibular joint (TMJ) problems, interdental spacing and overbite).

#### Inclusion criteria

Studies that included above interventions and outcomes and addressed the pre-specified outcomes were eligible for this SR.

#### Exclusion criteria

The following study designs: case-control, cross-sectional and cohort studies; case-series and case reports; other SRs; analytical and narrative reviews and different types of animal studies that were not eligible for inclusion, were excluded.

#### Search strategy

All relevant databases were searched: Medline, Cochrane Central Register of Controlled Trials, EMBASE, CINAHL, Science Direct, ProQuest, Science Journals, Scopus, PsycINFO, ClinicalTrials.gov, WHO ICTRP, TRIP and PACTR. Further hand-searching was conducted including citations from reference lists of retrieved studies (PEARLing searches) for additional references [59]. Where data were missing and full texts unavailable, these unclear reports were clarified by contacting authors or research institutes. Efforts were made to obtain English versions of studies reported in other languages either by requesting English versions from authors or using language experts to translate key findings. Authors were also contacted for unpublished reports or conference proceedings, where it was needed. Where registries were available for on-going studies, these were included as well and experts in the field of research related to the SDA were contacted.

Key terms were combined using Boolean operators and search strategies for each database were developed using the database specific functions [59]. Medical subject headings were applied in databases which allowed this function [59]. A wide search strategy was developed and modified according to the requirements of the different databases to ensure no eligible studies were excluded and an example includes the following:

(shortened dental arch OR shortened dental arches) AND (Clinical Trial OR Comparative Study OR Evaluation Studies OR Randomized Controlled Trial OR clinical trial) AND 1980/01/01-2014/12/31).

#### Search limits

Databases were searched for articles of over a period of three decades from 1980 to April 2014. The limits included in the search strategy were: human studies, adult patients and randomized and non-randomized controlled clinical trials.

#### Selection methods

Two review authors (SK and AM) independently screened titles and abstracts from the electronic searches to select potentially relevant studies using a predetermined eligibility form based on the inclusion criteria [59]. Full text articles of potential studies were then retrieved and re-assessed for eligibility. Each article was scrutinized to ensure that multiple publications from the same study were included only once. Where eligibility was unclear, clarification was sought from the trial authors and the corresponding articles were re-assessed. Differences between the eligibility results were resolved by consulting the other review authors (UMEC and RO). Studies that did not meet the inclusion criteria were excluded and the reasons for exclusion were reported. Data extraction for the selected studies was completed by the principal researcher (SK) using a specially designed pre-piloted data extraction form for this SR [59]. All disagreements regarding this process were resolved through discussion with the other review authors (AM, UMEC and RO).

#### Qualitative analysis

The quality of the studies included for this SR were evaluated for any risk of bias by researchers (SK and AM) using the Cochrane Risk of Bias tool and as described in the Cochrane Handbook for Systematic Reviews of Interventions [59]. The assessment was done across the following six components: random sequence generation, allocation concealment, blinding, incomplete outcome data, selective reporting, and other bias. Each of these were judged as ‘yes’, ‘no’, or ‘unclear’ corresponding to low, high, or unclear risk of bias respectively. Where information in the articles was insufficient for making the judgements, trial authors were contacted for clarification. Disagreements were resolved through discussion with other review authors. Results of risk of bias were summarised in a risk of bias table. In addition, GRADE assessments were completed by the researchers (SK and AM) for each clinical trial and these were used to grade the evidence and strength of recommendations for clinical intervention (where possible) using the GRADE Profiler system [60]. These are reported in the summary of findings tables.

#### Data synthesis and management

Results were reported separately for the following three comparisons: 1) FDP versus RPDP; 2) RPDP versus no treatment (SDA); 3) SDA versus CDA. No imputation of missing data was carried out and study authors were requested to provide any missing data. Available case analysis was applied where data were missing. Risk ratios with corresponding 95% confidence intervals were calculated for dichotomous outcomes using Review Manager 5 software. Although a meta-analysis of outcomes across study results had been anticipated, the included studies reported outcomes in different forms that could not be pooled in a meta-analysis. Consequently, results for individual studies were reported separately.

## Results

**Figure 1:**
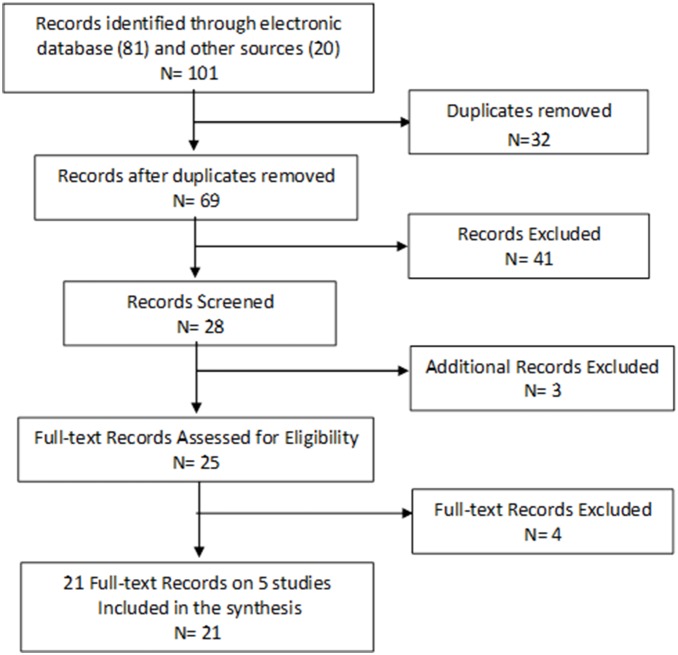
Prisma Flow Chart of Study Selection.

The search strategy identified a total of 101 citations (Figure 1): electronic databases yielded 81 and 20 were from reference lists of retrieved articles (that is, through PEARLing searches). A total of 32 duplicate records were removed, leaving 69 citations which were assessed for eligibility. After reading titles and abstracts, a total of 41 records were excluded and the full-text of the remaining 28 records was retrieved. A further 7 records were excluded after reading the full-text reports, leaving the remaining 21 records as included studies for the SR (Figure 1). Only four RCTs and one CT were used for this review, but outcomes were reported in the retrieved 21 records [14], [31]–[38], [40]–[48], [53]–[55]. No on-going studies were found and no eligible studies were excluded for failure to report the reviewer’s pre-specified outcomes.

### Study characteristics

The studies were grouped according to types of interventions into the following comparisons:

*Comparison 1*: FDPs versus RPDPs for SDAs in the lower jaw.

Two included studies from the UK and Denmark assessed comparison 1 [31]–[38].

*Comparison 2*: RPDPs versus no treatment (SDA).

Two studies from Germany and Ireland assessed comparison 2 [40]–[48].

*Comparison 3*: SDA versus CDA.

Only one study from the Netherlands assessed comparison 3 [14], [53]–[55].

#### Characteristics of included studies

The study characteristics of the four RCTs and 1 CT included in this SR are summarized according to types of study, population characteristics, types of interventions and the follow-up periods and these are specified on Table 1 [14], [31]–[38], [40]–[48], [53]–[55].

**Table 1:** Table of Included Studies.

Study Details	Methods	Participants	Interventions	Outcomes	Notes
*Author*: Budtz-Jorgensenand Isidor (31–33)	*Duration of* *trial*: 5 years	*Sample*:Total N = 53	*Intervention*:FDP (N = 27)	*Outcomes*: Caries;Prosthetic condition;periodontal conditions(PI/GI) and	Study approval by EthicsBoard was not recorded
*Country*:Denmark	*Assessment* *periods*: 1 and2 months; 2 and 5years	*Age*: 61–83 yrs. (Meanage: 69) *Gender*: 28Females; 25 Males	*Control*:RPDP (N = 26)	Masticatory system(TMJ) and patient opinion.	No verification
*Study Design*:CT		*Country and Setting*:Denmark, UniversityHospital		Outcomes were not dividedinto primary or secondary	
*Author*: Witteret al (14, 53–55)	*Duration of* *trial*: 9 years	*Sample*:Total N = 146	*Intervention*:SDA (N = 74)	*Outcomes*: Interdentalspacing; periodontalsupport and	Study approved byUniversity NijmegenEthics Board.
*Country*:Netherlands	*Assessment* *periods*:Baseline and 3,6 and 9 years	*Age*: Mean −36.2 yrs.for CDA; Mean –40.5yrs. for SDA *Gender*:82 Females;64 Males	*Control*:CDA (N = 72)	Occlusal contact; Overbite;occlusal wear and TMJproblems	Informed Consent frompatients was obtained.
*Study Design:*CT		*Country and Setting*:Netherlands,Nijmegen Clinic		Outcomes were not dividedinto primary or secondary	
*Author*:Jepson et al(34–37)	*Duration of* *trial*: 2 and 5years	*Sample*:Total N = 60	*Intervention*:FDP (N = 30)	*Primary*: Survival ofprosthesis; Influence of dietand nutrient intake	Study approval receivedfrom Ethics Board.
*Country*:UnitedKingdom(UK)	*Assessment* *periods*: 3months; 1, 2and 5 years	*Age*: 39–81 yrs.(Mean age: 67) *Gender*:35 Females;25 Males	*Control*: RPDP (N = 30)	*Secondary*: Caries; Periodontalstatus; patient satisfaction	Informed Consent frompatients obtained.
*Study* *Design*:RCT		*Country and Setting*: UK, Newcastle Dental Hospital			Power calculations werecompleted
*Author*:Walters et al(40–46)	*Duration of* *trial*: 3 year	*Sample*: Total N = 215(pilot sample incl. inmain study)	*Intervention*:SDA (N = 106)	*Primary*: First tooth loss	Study approved byInstitutional Ethics ReviewBoard
*Country*:Germany	*Assessment* *periods*: 4–8wks.; 6 monthsand 1, 2, 3, 4and 5 years	*Age*: 35 yrs. + (Meanage: 59)*Gender*:107 Females and108 Males	*Control*: RPDP(N = 109)	*Secondary*: 2^nd^ tooth loss;caries; survival oftreatment; oral healthrelated quality oflife; tooth mobility;PI; GI and TMJProblems	Power calculations werecompleted
		*Country and Setting*:Germany, UniversityHospitals			
*Author*:McKennaet al (47–48)	*Duration of trial*:1 year	*Sample*:Total N = 44	*Intervention*:RPDP (N = 21)	*Primary*: Oral healthrelated quality ofLife; Nutritionalstatus	Study approved by CorkUniversity’s EthicsReview Board
*Country*:Ireland	*Assessment* *periods*: Baselineand 1 month	*Age*: 65–82 yrs.(Mean age: 68)*Gender*: 28Females; 16 Males	*Control*:RBB/FDP (N = 23)	*Secondary*: cost-effectivenessof two treatments	Power calculationscompleted:Estimated that one treatment wasnot worse than the other
*Study* *Design:* RCT		*Country and Setting*:Ireland, UniversityHospitals			

KEY:

RCT–randomized controlled trial.

CT–Clinical Trial.

SDA–shortened dental arch.

CDA–complete dental arch.

FDP–fixed dental prosthesis.

RBB–resin-bonded bridge.

RPDP–removable partial denture/dental prosthesis.

PI–plaque index.

GI–gingival index.

TMJ–temporomandibular joint.

#### Qualitative analysis

**Figure 2:**
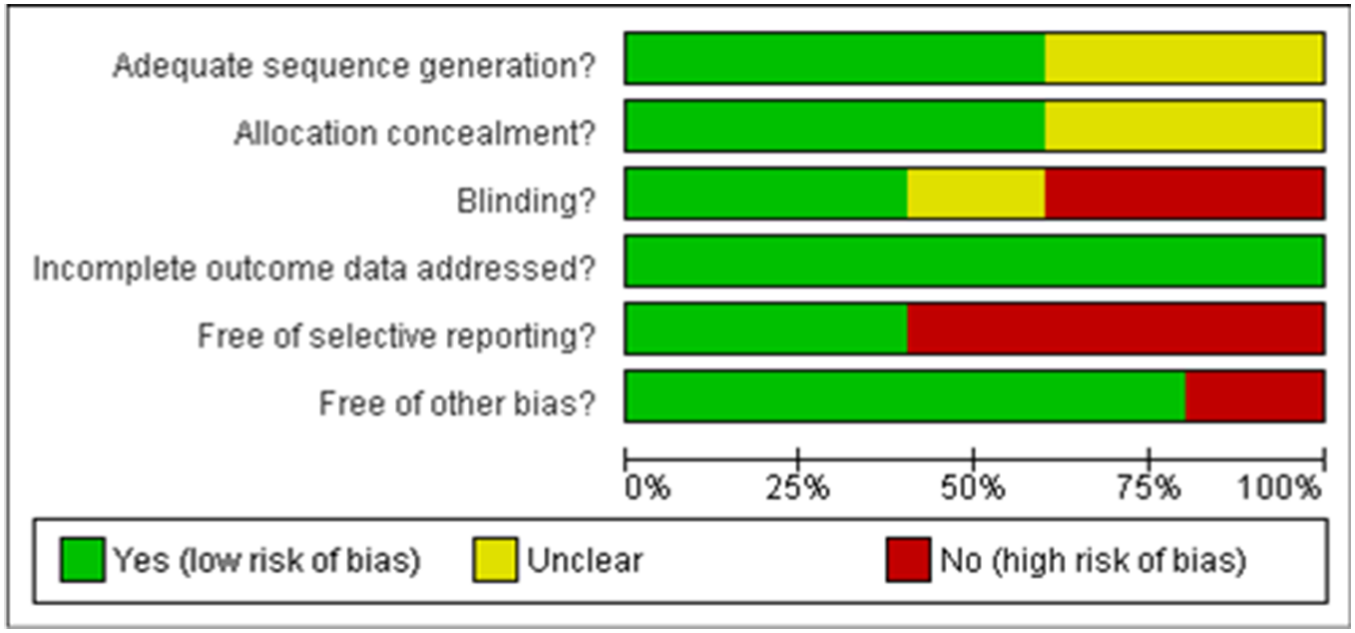
Risk of bias graph: review authors’ judgements about each risk of bias item presented as percentages across all included studies.

Table 2 specifies the quality assessment of the included studies and these are summarized in the ‘risk of bias table’ and ‘risk of bias graph’ where judgements are categorized to indicate a low, high, or unclear risk of bias (Figure 2) following the Cochrane guidelines [59]. Below we give a detailed explanation of these results:

**Table 2:** Risk of Bias Table.

Study	Budtz-Jorgensen(31–33)	Witter et al(14, 51–53)	Jepson et al(34–38)	Walter et al(40–46)	Mc Kenna et al(47–48)
**Random Sequence Generation**(Selection bias)	Unclear	Unclear	Yes	Yes	Yes
**Allocation Concealment**(Selection bias)	Unclear	Unclear	Yes	Yes	Yes
**Blinding**(Detection and Performance bias)	No	Unclear	Yes	No	Yes
**Incomplete Outcome Assessment**(Attrition bias)	Yes	Yes	Yes	Yes	Yes
**Free of Selective Reporting**(Reporting bias)	No	No	Yes	No	Yes
**Free of Other Bias**	No	Yes	Yes	Yes	Yes

“Yes” indicates a low risk of bias, “No” indicates a high risk of bias, and “Unclear” indicates either a lack of information or uncertainty over the potential for bias.

Sequence Generation: Three of the five trials were reported as having been randomised. For sequence generation**,** two clinical trials used computer-generated numbers and a third trial used randomly permuted block randomisation for generating the allocation sequence, which we judged as having a low risk of bias [34]–[38], [40]–[48]. The Witter et al (2001) clinical trial invited subjects to join the department for a study, and no attempt was made to randomise patients, thus it is judged as having a high risk of bias [14], [53]–[55]. The Budtz-Jorgensen and Isidor (1987) trial did not mention how the sequence was generated and provided insufficient information to enable us to judge whether there was a high or low risk of bias, and we thus rated it as having an unclear risk of bias [31]–[33].

Allocation Concealment: The Moynihan et al (2000), Wolfart et al (2005) and Mc Kenna (2012) studies are described as having a low risk of bias for allocation concealment, as they indicated that the clinician was not involved in the allocation and that concealment was warranted following a central randomisation process after patient enrolment [34]–[38], [40]–[48]. For the Budtz-Jorgensen and Isidor (1987) and Witter et al (2001) studies, there is no indication as to how intervention allocation was concealed and these were judged as having an unclear risk of bias [14], [31]–[33], [53]–[55].

Blinding: The Moynihan et al (2000) study was referred to as a double blinded study with the clinician blinded to allocation of intervention and statistician being blinded to treatment and thus it is judged as having a low risk of bias [34]–[38]. The Witter et al (2001) study can be considered as a single blinded study because evaluation of outcomes was completed by a calibrated observer at all intervals, but it was not stated as such, thus it is judged as having an unclear risk of bias [14], [53]–[55]. Mc Kenna (2012) indicated that the researcher was not involved in the intervention allocation, making it a single-blinded study, thus it is judged as having a low risk of bias [47]–[48]. The Wolfart et al (2005) study indicated that it was impossible to blind the dentist and patient due to discrepancies of the treatments; thus it was judged as having a high risk of bias, whereas Budtz-Jorgensen and Isidor (1987) provided insufficient information related to blinding and it was regarded as having an unclear risk of bias [31]–[33], [40]–[46].

Incomplete Outcome Data: Analyses for the Moynihan et al (2000), Wolfart et al (2005) and Mc Kenna (2012) studies were conducted on the “intention-to-treat” (ITT) principle; and the studies reported proportionate numbers of losses to follow-up (which were small) and some having no losses between the intervention and control [34]–[38], [40]–[48]. Witter et al (2001) indicated that regression models accounted for the subjects lost during the study [53]. Thus, all 4 studies above were judged as having a low risk of bias [34]–[38], [40]–[46], [53]–[55]. On the other hand, Budtz-Jorgensen and Isidor (1987) did not indicate and specify how the analysis was completed, but all pre-specified outcomes were reported, and the number of losses to follow-up was small, thus it was judged as having a low risk of bias [31]–[33].

Selective Reporting: All studies were registered and approved with their respective Review boards [14], [31]–[38], [40]–[48], [48], [53]–[55]. The protocol for the Wolfart et al (2005) study was published (41). In the Budtz-Jorgensen and Isidor (1987) and Witter et al (2001) studies all outcomes were reported but outcomes were not pre-specified as primary or secondary outcomes [14], [31]–[33], [53]–[55]. Both these studies were thus judged as having a high risk of bias. The three remaining RCTs specified the outcomes as primary and secondary and reported these as such, thus these were judged as having a low risk of bias [34]–[38], [40]–[46]. All the included studies except the Wolfart et al (2005) study reported all their pre-specified outcomes in subsequent publications [14], [31]–[38], [40]–[48], [53]–[55].

Other potential sources of bias: No other sources of bias were detected with four of the five included studies. The Budtz-Jorgensen and Isidor (1987) study was judged as having high risk of bias because there were six patients who did not wear the RPDP at all during the study [32]–[33].

### Effects of interventions

See: Summary of findings for the main comparisons of functional outcomes and patient satisfaction with FDPs compared to RPDPs in treating patients with SDAs (Table 3); Summary of findings for SDA patients treated with RPDPs compared to no treatment (Table 4) [59].

**Table 3:** COMPARISON 1: FDP versus RPDP for Treated and untreated Shortened Dental Arches (31–38).

**Patient or population:** patients with Treated and untreated Shortened Dental Arches						
**Settings:** Hospital Setting						
**Intervention:** FDP versus RPDP						
**Outcomes**	Illustrative comparative risks* **(95% CI)**		**Relative effect**	**No of Participants** **(studies)**	**Quality of the** **evidence**	**Comments**
	Assumed risk	Corresponding risk	**(95% CI)**		**(GRADE)**	
	**Control**	**FDP versus RPDP**				
**Number of patients with** **satisfactory occlusion**	**Study population**		**RR 1.16**(0.9 to 1.48)	53 (1 study)	⊕⊖⊖⊖**very low** ^1,2^	
	**769 per 1000**	**892 per 1000**(692 to 1000)				
	**Moderate**					
	**769 per 1000**	**892 per 1000**(692 to 1000)				
**Number of patients with** **no caries experience**	**Study population**		**RR 1.89**(1.09 to 3.3)	50 (1 study)	⊕⊕⊕⊖**moderate** ^2^	
	**391 per 1000**	**740 per 1000**(427 to 1000)				
	**Moderate**					
	**391 per 1000**	**739 per 1000**(426 to 1000)				
**Number of patients** **showing TMJ** **dysfunction**	**Study population**		**RR 0.64**(0.36 to 1.16)	53 (1 study)	⊕⊖⊖⊖**very low** ^1,2^	
	**577 per 1000**	**369 per 1000**(208 to 669)				
	**Moderate**					
	**577 per 1000**	**369 per 1000**(208 to 669)				
**Survival of Intervention**	**Study population**		**HR 0.59**(0.27 to 1.29)	60 (1 study)	⊕⊕⊕⊖**moderate** ^2^	
	See comment	See comment				
	**Moderate**					
**Patient Satisfaction**	**Study population**		Not estimable^3^	52 (1 study)	⊕⊕⊕⊖**moderate** ^2^	
	See comment	See comment				
	**Moderate**					

EXPLANATION OF TABLE ABOVE:*The basis for the **assumed risk** (e.g. the median control group risk across studies) is provided in footnotes. The **corresponding risk** (and its 95% confidence interval) is based on the assumed risk in the comparison group and the **relative effect** of the intervention (and its 95% CI). **KEY: CI:** Confidence interval; **RR:** Risk ratio; **HR:** Hazard ratio.

***Explanation for the GRADE Working Group QUALITY of evidence*** *:* *High quality*: Further research is very unlikely to change our confidence in the estimate of effect. **Moderate quality:** Further research is likely to have an important impact on our confidence in the estimate of effect and may change the estimate. *Low quality*: Further research is very likely to have an important impact on our confidence in the estimate of effect and is likely to change the estimate. **Very low quality:** We are very uncertain about the estimate.

***REASONS for the QUALITY of the Evidence:*** **** **^1^**High risk of bias for blinding, selective reporting bias and other bias; **^2^**Small sample size; **^3^**No significant difference (p = 0.092).

**Table 4:** COMPARISON 2: RPD versus SDA for Treated and untreated Shortened Dental Arches (40–48).

**Patient or population:** patients with Treated and untreated Shortened Dental Arches							
**Settings:** Hospital setting							
**Intervention:** RPD versus SDA							
**Outcomes**	Illustrative comparative risks* **(95% CI)**			**Relative effect** **(95% CI)**	**No of Participants** **(studies)**	**Quality of the** **evidence** **(GRADE)**	**Comments**
	Assumed risk	Corresponding risk					
	**Control**	**RPD versus SDA**					
**Change in MNA scores**		The mean change inMNA scores in theintervention groupswas **0.03 lower**(1.35 lower to 1.29 higher)			42 (1 study)	⊕⊕⊕⊖**moderate** ^1^	
**Number of patients with** **first tooth loss in 38 months**	**Study population**			**0.83**(0.74 to 0.91)	150 (1 study)	⊕⊕⊖⊖**low^2,3^**	
	**130 per 1000**	**108 per 1000**(97 to 119)					
	**Moderate**						
	**130 per 1000**	**108 per 1000**(96 to 118)					
**Patient Satisfaction**	**Study population**			Not estimable^4^	215 (1 study)	See comment	
	See comment		See comment				
	**Moderate**						

EXPLANATION OF TABLE ABOVE:*The basis for the **assumed risk** (e.g. the median control group risk across studies) is provided in footnotes. The **corresponding risk** (and its 95% confidence interval) is based on the assumed risk in the comparison group and the **relative effect** of the intervention (and its 95% CI). **KEY: CI:** Confidence interval; **RR:** Risk ratio;

***Explanation for the GRADE Working Group QUALITY of evidence*** *:* *High quality* **:** Further research is very unlikely to change our confidence in the estimate of effect. **Moderate quality:** Further research is likely to have an important impact on our confidence in the estimate of effect and may change the estimate. **Low quality:** Further research is very likely to have an important impact on our confidence in the estimate of effect and is likely to change the estimate. *Very low quality*: We are very uncertain about the estimate.

***REASONS for the QUALITY of the Evidence*** **: ^1^**Small sample size; **^2^**High risk of bias for blinding and selective reporting bias; **^3^**Wide confidence interval- the 95% CI includes both null effect and appreciable harm and **^4^**No significant changes were reported for the Irish study. For the German study: Significant differences were seen at baseline (27.0; p<0.0001) and 1 year on (13.0; p<0.0002) for the RPDP group and a significant change in impacts (19.0; p<0.05) were observed only at baseline for the SDA group.

#### Comparison 1: Fixed Denture Prosthesis vs Removable Partial Denture Prosthesis

Primary Outcomes: 1. Functional Outcomes: *Occlusion:* Budtz-Jorgensen and Isidor (1987) showed no significant difference in the number of patients with satisfactory occlusion during the 2-year period after treatment between the FDP and RPDP groups (RR 1.16, 95%CI: 0.90 to 1.48, 53 participants) [31].

2. Survival: Thomason et al (2007) reported time to survival for the restoration of the shortened dental arch but there was no significant difference between the FDP and RPDP groups (Hazard Ratio 0.59, 95%CI: 0.27 to 1.29, 60 participants) [37].

Secondary Outcomes: 1. Patient Satisfaction: This outcome was only reported by Jepson et al (2003) but there was no significant difference in median satisfaction scores at 1 year after treatment between the FDP and RPDP groups (p = 0.092 as reported by authors, 52 participants: Table 5) [36].

**Table 5:** Summary satisfaction scores for the UK-based study at 1 year (a lower score indicates more satisfaction).

Group	N	Median (baseline)	Median (1 year)	p-value per group	p-value between groups
FDP (Intervention)	26	18	11	<0.001	0.092
RPDP (Control)	26	16.5	13	0.009	

FDP =  Fixed dental prosthesis; RPDP = Removable partial denture/dental prosthesis (34–38).

2. Harmful Effects: (caries; tooth loss; periodontal status, plaque index, gingival index; TMJ problems; interdental spacing; overbite).

*Caries:* Both studies are in agreement regarding the development of caries lesions with FDPs and RPDPs where: Jepson et al (2001) found that treatment with FDPs showed a significant increase in number of patients with no caries experience compared to the RPDP patients (RR 1.89, 95% CI: 1.09 to 3.30, 50 participants) [35]. Similarly, Isidor and Budtz-Jorgensen (1990) observed 22 dental carious lesions in the RPDP group compared with only two lesions in the FDP group; however we could not calculate a treatment effect since the respective number of patients was not reported. Our unit of analysis was individual patients and not individual teeth [33].

The following effects were only reported for the Budtz-Jorgensen and Isidor study (33):

*TMJ dysfunction:* Isidor and Budtz-Jorgensen (1990) found no significant difference in the number of patients showing TMJ dysfunction between the FDP and RPDP groups (RR 0.64, 95%CI: 0.36 to 1.16, 53 participants) [33].

*Tooth Loss:* In the Isidor and Budtz-Jorgensen (1990) study, 11 teeth were extracted in the RPDP group compared with only one tooth in the FDP group during the five years of observation. However, no treatment effect could be calculated because the respective numbers of patients were not reported [33].

*Plaque Index:* Isidor and Budtz-Jorgensen (1990) reported the mean plaque index ranging from 0.4 to 0.7 in the FDP group and from 0.7 to 1.0 in the RPDP group; the difference between the two groups was significant (p<0.05) during the first two years of examination as reported by study authors [33].

*Gingival Index:* Isidor and Budtz-Jorgensen (1990) indicated that the mean gingival index was always higher in the RPDP than in the FDP group, the difference being significant (p<0.05) at the 12-, 18-, 36-, and 48-month examinations [33].

#### Comparison 2: Removable Partial Denture Prosthesis versus no treatment (SDA)

Primary Outcomes: 1. Functional outcomes: *Mini Nutritional Assessment (MNA):* Mc Kenna (2012) reported the change in MNA scores from baseline to final (month 1) for the two treatment groups and these results are summarised in Table 6 [47]. The values in the table were used to calculate a treatment effect which showed no significant difference in the change in MNA score between the RPDP and SDA treatment groups (MD −0.03, 95%CI: –1.35 to 1.29, 42 participants: Table 6). A higher MNA score indicates better nutrition effect.

**Table 6:** Change in MNA scores for the Irish study.

Group	n	Baseline MNAscore average	Final MNA scoreaverage	p-value per group	Calculated SD of change
RPDP	21	23.65	24.75	0.03	2.15
SDA	21	23.24	24.37	0.03	2.21

MNA = Mini nutritional assessment; SD = Standard Deviation; RPDP = Removable partial denture/dental prosthesis; SDA = Shortened dental arch (47–48).

2. Survival: This outcome was not reported in the two studies assessing this comparison.

Secondary Outcomes: 1. Patient satisfaction: This outcome was measured using different tools for both the Mc Kenna (2012) and Wolfart studies (2012), but the time periods from baseline to the end of studies were significantly different, thus indicating differences in final outcomes.

*Oral Health Related Quality of Life (OHRQoL):* Mc Kenna (2012) reported a non-significant difference in the OHRQoL scores from baseline to the end of treatment (month 1) for the two treatment groups (Table 7) [47]. The author used the oral health impact profile (OHIP-14) to give a score ranging from 0 (minimum) to 56 (maximum). A high score indicated a poor OHRQoL with low scales indicating good OHRQoL. However, no treatment effect could be calculated to compare the change in the OHIP-14 scores between the two treatment groups because standard deviations of change were not given and also because exact p-values were not reported.

**Table 7:** Change in OHIP-14 scores for the Irish study.

Group	n	Baseline OHIP-14 scoreaverage	Final OHIP-14 scoreaverage	p-value per group
RPDP	21	12.4	3.3	<0.001
SDA	21	11.4	1.8	<0.001

OHIP = Oral health impact Profile; RPDP = Removable partial denture/dental prosthesis; SDA = Shortened dental arch (47–48).

For the Wolfart et al study (2012), the median OHIP-49 scores for pre-treatment, baseline, 1 and 5 years follow-up showed significant reduction of impacts (p<0.05). Before treatment, the median OHIP-49 total score was 38.0 for the RPDP group and 40.0 for the SDA group. Most significant reductions occurred at baseline (27.0; p<0.0001) and 1 year on (13.0; p<0.0002) for the RPDP group (compared to the Mc Kenna study after 1 month). For the SDA group, a significant change in impacts (19.0; p<0.05) were observed only at baseline, no further significant changes were reported [45].

2. Harmful Effects: (caries; tooth loss; periodontal status, plaque index, gingival index; TMJ problems; interdental spacing; overbite).

*Tooth loss:* The Walter et al study (2012) showed no significant difference in the number of patients experiencing first tooth loss within 38 months of observation after treatment between the RPDP and SDA groups (RR 1.23, 95%CI: 0.56 to 2.70, 150 participants) [44]. The respective Kaplan-Meier survival rates at 38 months were 0.83 (95%CI: 0.74 to 0.91) in the RPDP group and 0.86 (95%CI: 0.78 to 0.95) in the SDA group, the difference is not significant (as reported by study authors) [44].

#### Comparison 3: Shortened Dental Arches (SDA) versus Complete Dental Arches (CDA)

Primary Outcomes: 1. Functional outcomes:

*Occlusal contact:* Witter et al, (2001) reported that a significantly higher percent (73%, 95%CI: 67–80%) of teeth in the anterior region had occlusal contact in intercuspal position of the SDA group compared with the CDA group (62%, 95%CI: 55–69%) (p<0.05) [53]. No treatment effect could be calculated because the number of patients per group was not specified [53].

*Occlusal tooth wear:* Witter et al (1994) reported the mean occlusal tooth wear scores using transformed values for subjects of 40 years of age [55]. However, no significant differences between the SDA subgroups [means (SD) ranging from 1.1(0.1) to 1.6(0.1)] and the CDA group [means (SD) of 1.4(0.0) and 1.5(0.0)] were found when comparing the means of the scores for the upper and for the lower anterior regions. Similarly for the premolar regions, no significant differences were found between the SDA subgroups [mean (SD) scores 0.7(0.1) to 1.0(0.1)] and the CDA group [mean (SD) score 0.9(0.1)]. No treatment effect could be calculated because the respective number of patients was not reported.

2. Survival: This outcome was not reported in the one study assessing this comparison.

Secondary Outcomes: 1. Patient satisfaction: This outcome was not reported in the one study assessing this comparison.

2. Harmful Effects: (caries; tooth loss; periodontal status, plaque index, gingival index; TMJ problems; interdental spacing; overbite).

*Interdental spacing:* Witter et al (1994) described a comparison of the mean scores of interdental spacing per region [55]. According to the authors, the premolar regions of the SDA subgroups had significantly higher means [mean (SD): 0.4(0.1) and 0.5(0.1)] than the CDA group [mean (SD): 0.1(0), p<0.01 as reported by authors]. For the anterior regions, the spacing was not significantly different for SDA [mean (SD) range from 0.2(0.1) to 0.5(0.1)]; CDA group [mean (SD) range from 0.1(0.0) to 0.3(0.1)]. They also reported that spacing remained the same in all regions over time in the SDA group [55]. No treatment effect could be calculated because the results were given per region and also because the respective number of patients were not specified in the results.

*Overbite:* Witter et al (1994) stated this outcome only for some subgroups but did not compare their results between the SDA and CDA groups [55]. Therefore we could not calculate a treatment effect.

*Periodontal support:* Witter et al (1994) described the mean relative bone heights using transformed values for subjects of 40 years of age [55]. The authors reported that maxillary premolars and mandibular second premolars in the SDA subgroups showed significantly lower mean bone height scores than those in the CDA group, whereas mandibular first premolars did not differ. The values reported were not sufficient for the calculation of a treatment effect.

*TMJ problems:* The Witter et al study (2007) indicated that patients with SDAs (65–79%) had similar prevalence, severity and changes in signs and symptoms related to the TMJ as patients with CDAs (70–75%) [54].

*Excluded study characteristics:* All non-RCTs and reviews were excluded from this SR. Other SRs and summary articles were viewed as potentially included studies, but these were however later not considered for inclusion (Table 8).

**Table 8:** Excluded studies, with reasons for exclusion.

Study	Reasons for exclusion
Abt, Carr and Worthington (57)	A systematic review
	Focused on treatment options for all types of partially dentate patients
	Did not specifically focus on the interventions for SDAs
	SDA was considered as only one treatment option
Fueki et al (56)	A systematic review completed in Japan
	Included different study designs
	All the RCTs included in this review were used for the present review as well. But other RCTs were included for the present SR
	The analysis for this SR is different to that of the present SR
Faggion (58)	A systematic review
	Intention was to include RCTs and CTs, but a prospective study was included
	All RCTs used for this SR was included in the present review with the inclusion of other RCTs
	Outcomes that were not reported in this SR has been included in the present review
	Focus of this paper was the GRADE assessment completed
Emami and Feine: 2010 (62)	Is a summary of a clinical trial completed on this SDA subject. Above RCT has been included in this review
Gotfredsen and Walls (8)	Is a SR of the literature related to the SDA topic
	Similar outcomes as addressed in this SR
	Different study design types were included
	SR concluded the acceptable level of oral function obtained with 20 natural teeth (which is line with the WHO goal for the year 2000)

KEY:

SDA: shortened dental arch.

RCT: randomized controlled trial.

CT: clinical trial.

SR: systematic review.

GRADE: Grading of Recommendations Assessment, Development and Evaluation.

WHO: World Health Organization.

## Discussion

The focus of this review was the classic SDA, irrespective of whether it occurred naturally or was created by means of a FDP. An exhaustive and comprehensive search yielded four RCTs and 1 CT that were included [14], [31]–[38], [40]–[48], [53]–[55]:

Jepson et al (2001) is in agreement with the research conducted by Isidor and Budtz-Jorgensen (1987, 1990) regarding an increase in caries incidence as reported 2 and 5 years post treatment [33], [35], [38]. In addition, the increase in caries incidence for the RPDP group also concurred with the research of Bergman et al, (1964), cited in Budtz-Jorgensen (1990) [32].

Survival of fixed bridges 5 years post study was similar to other trials [30]–[32], [37]–[38]. RPDP patients chose not to wear RPDPs which was similar to other studies [31]–[32], [37]–[38]. For patient satisfaction, the small sample size does not allow us to generalize our results to other settings, thus it is advised to conduct these studies amongst different populations.

For the Wolfart et al study (2010): Post hoc power calculations implied that the pilot sample size was too small to generalize results and for comparison to other studies [40]–[43]. The larger study results are free of bias with a large enough sample due to it being a multi-centre study. While it reduced the bias, it still could not be generalized to patients that are different to the study sample. For the patient satisfaction outcome, the summary scores of the pilot study were similar to another German study (John and Micheelis, 2003, cited in Walter et al (2012) [45]. For temporomandibular disease (TMD) pain scores, the instrument used in other studies was more reliable (Dworkin, 2002, cited in Walter et al (2012) [44]. Tooth loss as a primary outcome is questioned due to extended time periods, thus it was advised to use caries and periodontal attachment loss as outcomes instead [44].

The Mc Kenna study (2012), which is the most recently conducted RCT; the results are similar to other RCTs completed in the past, where small sample sizes would not necessarily show a significant difference between interventions given the follow-up period [47]–[48]. In this case, follow-up after only one month of treatment was too short to show any difference between interventions [47]–[48]. But the cost-effectiveness reported with this RCT has been noted as researchers and clinicians are under the impression that the cost for FDPs far outweighs that of RPDP treatment [22], [39], [48]. And this has been in line with the findings of the Danish study published some years ago [32]–[33].

For the Witter et al study (2001), results were similar to other studies with regards to outcomes reported and the effect of outcomes on the dentition in the SDA group (tooth wear, TMJ effects) (Aukes, 1988; Mohl, 1988; Eliasson, 1997, cited in Witter et al (2001) [53].

The quality of the evidence is indicative of the integrity of the study and the research conducted. With reference to the quality assessment of the included studies, this has been described in detail above. More importantly, this quality is determined by the study designs. Study designs are graded according to the quality of evidence that they provide. Systematic reviews and RCTs are considered to be designs of the highest quality [59]–[60]. Within the different design groups, certain concessions can be made for those designs that do not follow the exact guidelines [59]–[60]. For instance RCTs can be downgraded if their risk of bias is high [59]–[60].

Only RCTs and CTs were however included in this systematic review which provides stronger evidence and increases the strength of the recommendations [59]–[60]. After completing the quality assessment (using the GRADE approach) of the included studies, it clearly showed that some of the studies had not followed the exact guidelines for RCTs, but nevertheless had the features thereof [59]–[60]. These can be regarded as downgraded RCTs (Tables 3–4). These downgraded RCTs did not use randomization, allocation concealment or blinding, and failed to specify the outcomes as primary or secondary. These downgraded RCTs could thus affect the quality of evidence only slightly [59]–[60]. For example, the Budtz-Jorgensen (1987, 1990) and Witter et al (2001) studies could be regarded as downgraded CTs [14], [31]–[33], [53]–[55], [60].

A meta-analysis could not be completed for this SR for the following reasons: Some of the outcomes for the SR (for example survival of intervention) were not reported by all the included studies; sufficient RCTs were not found related to SDAs; the outcomes were reported in so many different ways for each of the studies that a narrative approach for this review had to be adopted and not all outcomes are reported for the Wolfart et al (2005) study (and no correspondence was received when the authors were contacted). In addition, there was insufficient information reported by studies to allow us to combine continuous data using the mean difference (MD). The outcomes from the studies were thus grouped for this review.

For this SR, a systematic approach to the evaluation of the evidence obtained from the studies was adopted by the researchers and disagreements were resolved by discussion. The researchers highlighted the areas where bias could have been expected (Table 2). Study samples, settings, age categories, interventions and outcomes for the included studies were mostly similar, creating strong evidence (Table 1). Comparison between the groups of the different studies could be systematically recorded in the stipulated groups. And again, for this SR all potential sources were searched and reported. Most studies followed guidelines to protect against bias (some without making reference to the method followed) [14], [31]–[33], [53]–[55]. And this was assessed using the Cochrane’s risk of bias tool [59]. Since all the included studies in this SR were conducted in developed countries, our findings cannot be generalized to patients in all countries because cultural and socio-economic differences that exist between countries and within communities can influence patients’ reactions.

Other SRs were also conducted in the past ten years [8], [56]–[58], where researchers included studies with different study designs and not only RCTs. For the most current SR [57], the research question was so broad that the focus on the SDA was minimal, thus many of the data related specifically to SDAs were not even included in the analysis [57]. For this SR, only the British and German RCTs were mentioned and only the results of the pilot study for the German RCT was reported [57]. The authors concluded citing insufficient evidence to report a difference between RPDP and FDPs in the treatment of SDAs [57]. In addition, when evaluating the quality of the evidence of a systematic review, it is recommended that the GRADE approach should be used [60]. It is a method of evaluating the quality of evidence and strength of recommendations in healthcare, and thus provides the needed rigor and transparency when making specific recommendations [60].

### Quality of evidence

As stated above, the quality of evidence was assessed using the GRADE methodology for this SR (Tables 3 and 4). With the assessment, the small sample sizes seriously affected the imprecision, and the risk of bias was very serious with studies where no blinding and selective reporting was observed (Tables 3 and 4). From the combined effects, the overall quality of the assessment is regarded as being low (Tables 3 and 4). This implies that further research (as in conducting more RCTs) is likely to have an important impact on our confidence in the estimate of effect, and may change the estimate.

### Implications for practice

The SDA concept has been researched and used in industrialized countries and this review aimed to highlight its appropriateness and relevance for a developing country such as South Africa. A change in paradigm or thinking should be encouraged, even though results of clinical trials conducted in other countries may not necessarily be generalizable to South African populations. By regarding the research related to SDAs in a positive light (patient satisfaction, caries incidence, TMJ effects and tooth loss), this SR specifies that policy-makers and/or institutions should be encouraged and recommend its teaching and clinical implementation by students and clinicians. These are considered as instances where low-quality evidence can still make a strong recommendation due to the body of available evidence on SDAs.

### Implications for research

Sufficient RCTs related to SDAs were not found, and thus it would be advisable to conduct more randomized clinical trials. The RCTs were also conducted in European and Nordic countries and these results may not be generalizable to other context, due to substantial cross-cultural and socio-economic differences between countries. External validity or generalizability of studies conducted in other countries depends on: settings where studies were conducted; participants’ characteristics; interventions researched across studies; relevance of the endpoints achieved with each study; results obtained and their comparison to one another and the indirect/direct costs when conducting each study.

## Conclusions

The results from this SR related to SDAs as a treatment option were encouraging in terms of functioning, patient satisfaction and cost-effectiveness. However, only the Moynihan et al (2000) study reported on the primary outcome of survival of the SDA, and had this been determined by the other studies, it would have strengthened the recommendation of the SDA as a treatment option even further [34].

### Recommendations

The stronger the evidence, the stronger the recommendation for the implementation of the SDA as a treatment option for partially dentate patients. By using only high quality studies such as RCTs and CTs for this SR, it was expected that the results would be more reliable when making conclusions and recommendations. Nevertheless, any conclusion/s from such a SR can still be regarded in a positive light, even though the included studies had to be downgraded due to methodological errors [60]. It is also recommended that when conducting clinical trials, strict protocols need to be prepared and the reporting of the RCT should follow the CONSORT guidelines [61]. This could then be of great benefit to other researchers when critically appraising these clinical trials. More importantly, outcomes for the RCT have to be pre-specified and all should be reported so that future systematic reviews may be conducted with the inclusion of a meta-analysis, instead of a narrative report as needed to be done for this SR. Thus further research (as in conducting clinical trials) should be encouraged and for the different settings and contexts (for example developing countries) to create a comprehensive database related to SDAs.

## Supporting Information

Checklist S1 **PRISMA 2009 Checklist.**
(DOC)
